# RNASeq profiling of COVID19‐infected patients identified an EIF2AK2 inhibitor as a potent SARS‐CoV‐2 antiviral

**DOI:** 10.1002/ctm2.1098

**Published:** 2022-11-02

**Authors:** Sidharth Jain, Samantha Rego, Steven Park, Yiran Liu, Simone Parn, Kush Savsani, David S. Perlin, Sivanesan Dakshanamurthy

**Affiliations:** ^1^ Lombardi Comprehensive Cancer Center Georgetown University Medical Center Washington DC District of Columbia 20057 USA; ^2^ Georgetown College Georgetown University Washington DC District of Columbia 20057 USA; ^3^ Center for Discovery and Innovation Hackensack Meridian Health New Jersey 07110 USA; ^4^ Department of Biochemistry & Molecular Biology Georgetown University Medical Center Washington DC District of Columbia 20057 USA; ^5^ College of Arts & Science University of the District of Columbia Washington DC District of Columbia 20008 USA; ^6^ College of Humanities and Sciences Virginia Commonwealth University Richmond Virginia 23284 USA

Dear Editor,

In this study, we found gene expression changes in key genes involved in activating immune pathways and genes targeted by SARS‐CoV‐2 to interfere with normal host cell functioning. Notably, critical changes have been observed in Eukaryotic Translation Initiation Factor 2 Alpha Kinase 2 (EIF2AK2), which plays an important role in activating the interferon response and interfering with host cell translational machinery in SARS‐CoV‐2 infection,^1^, ^2^, ^3^ presenting a prospective therapeutic target. We demonstrated the therapeutic antiviral effect of the EIF2AK2 with its inhibitor compound C16, which showed strong antiviral potency in multiple antiviral assays.

We analysed the RNAseq data of human lung or respiratory tract samples from patients infected by COVID‐19. Our search of the Gene Expression Omnibus (GEO) database revealed four RNAseq data sets that met the inclusion criteria for the study. We identified 509 eligible patient RNAseq samples from GEO from GSE152075, GSE147057 and GSE150316 data sets (Supplementary Material), which were analysed using the Partek Flow genomics suite. Our differential gene expression analysis revealed several target genes and pathways that have been well described in SARS‐CoV‐2 pathogenesis, including the ACE2/TMPRSS2 surface receptors used for viral entry and interferon response and host translation machinery. Our drug–gene interaction analysis identified drugs, such as ribavirin and colchicine, which have been experimentally tested or are already in use to treat COVID‐19 patients,^4^, ^5^ validating the significance of our study.

We showed that the interferon signalling pathway was entirely upregulated, revealing many key genes responsible for activating the interferon response in SARS‐CoV‐2 infection (Figure 1A). At an individual gene‐level analysis, multiple ribosomal proteins were significantly downregulated in each data set (Figure 1B). Specifically, the expression of 40S and 60S ribosomal subunit proteins were significantly downregulated, suggesting that viral proteins interact with and modulate the expression of host translation machinery with SARS‐CoV‐2 infection. The eukaryotic initiation factor 2 (eIF2) signalling pathway was among the most downregulated pathways (Figure S4), which is shown to be involved in viral replication and transcription of viral genomic material. An important hub of this pathway is EIF2AK2, a kinase that is responsible for activating the inflammasome that was significantly upregulated in both analysed data sets (Figure 1). Along with suppressing ribosomal gene expression, EIF2AK2 acts as part of the cellular immune response to SARS‐CoV‐2, leading to downregulation and halt in host translation machinery. EIF2AK2 is involved in producing proinflammatory cytokines and inhibits the eIF2 pathway (Figure 1), suggesting that SARS‐CoV‐2 can impair host antiviral response with multiple mechanisms by acting on EIF2AK2. These findings led us to investigate the potential efficacy of the PKR (EIF2AK2) inhibitor to reverse the phenotype induced by SARS‐CoV‐2 infection.

**FIGURE 1:**
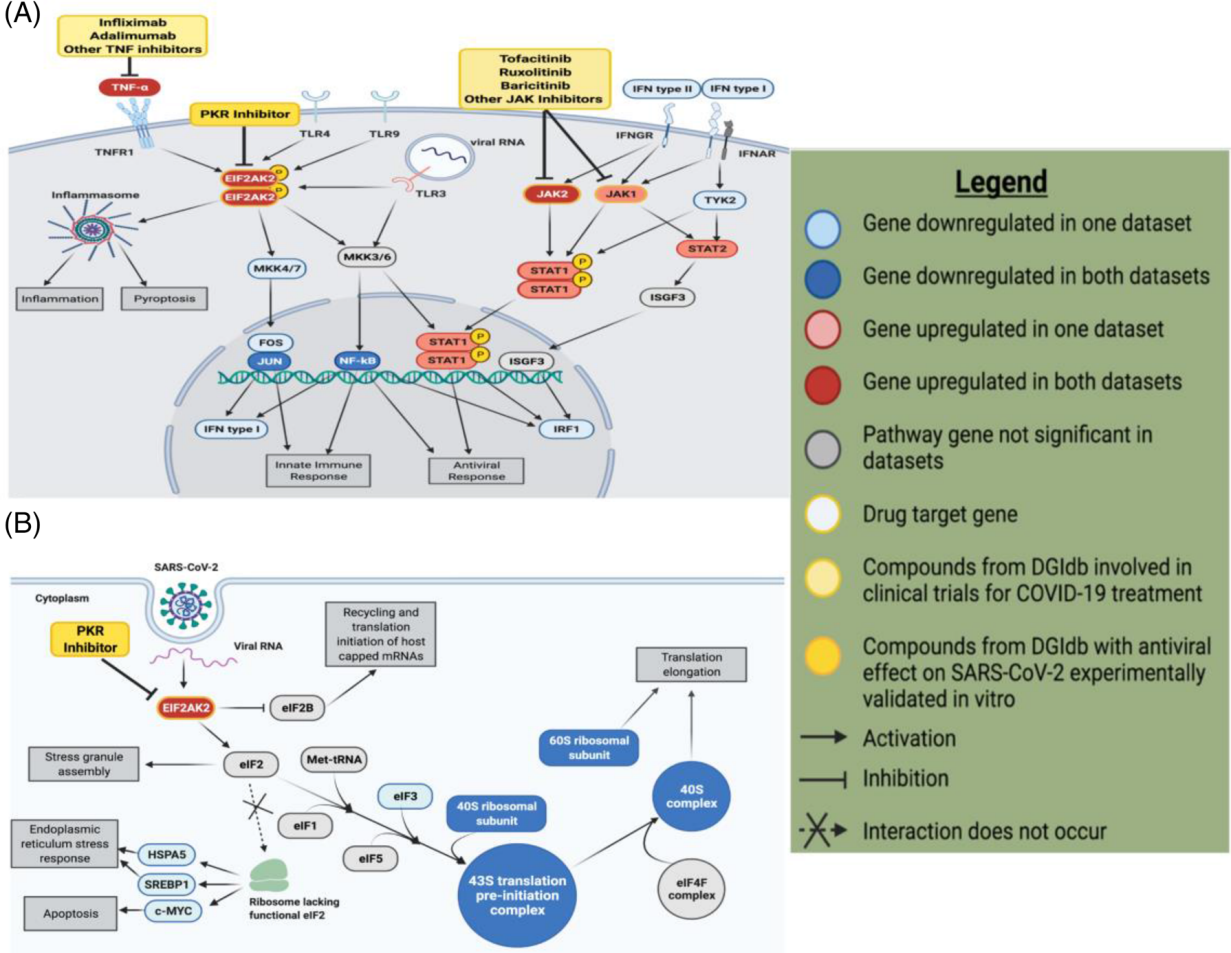
Differential gene and pathway expression analysis revealed key genes and potential drug targets involved in the host translation machinery with SARS‐CoV‐2 infection. (A) Upregulation of the interferon signalling pathway in SARS‐CoV‐2‐infected samples reveals many key genes and potential drug targets. This pathway figure was designed in BioRender.com based on the EIF2AK2 pathway diagram indicated as significantly represented in our data sets by IPA. Elements of the pathway not significant in the data sets analysed were excluded for clarity. Compounds identified from the DGIdb analysis whose effects on COVID‐19 have been suggested by other studies are shown modulating their respective targets. (B) Downregulation of key genes in the eIF2 signalling pathway reveals how SARS‐CoV‐2 interferes with host cell translation machinery to inhibit host protein synthesis. The eIF2 signalling pathway indicated as significant in our data sets by IPA was recreated in BioRender.com to highlight the elements of the pathway most significantly represented in our data. Genes that were not significantly differentially expressed in our data sets and did not interact with key genes were excluded from the figure for clarity. EIF2AK2 was the only potential drug target identified by DGIdb analysis within this pathway, shown modulated by PKR inhibitors. Figure legend was also designed in BioRender.com .

To further investigate the role of EIF2AK2 in the context of SARS‐CoV‐2 infection, an in vitro assay of EIF2AK2 inhibitor was performed. The EIF2AK2 inhibitor compound imidazolo‐oxindole C16, hereafter referred to as C16, has been shown to antagonise the kinase activity of PKR^6^ by binding to the ATP site. To support our findings, in multiple assays, we consistently demonstrated a substantial inhibitory effect of C16 on viral proliferation in SARS‐CoV‐2‐infected cells (Table 1). Compared to remdesivir, C16 showed relatively high potency in the 48H direct virus inhibition assay (EC_50_ = 1.25 μM) and the 72H ATP luminescence assay measuring cytopathic effect (EC_50_ = 2.96 μM) in A549+ACE2 cells (Figure 2A–C). Similarly, C16 antiviral activity was observed in the VeroE6 cells overexpressing TMPRSS2, a serine protease essential for SARS‐CoV2 cell entry (Figure 2D–F). The combination treatment of remdesivir + C16 showed a low to moderate synergism effect on VeroE6 cells with a reported EC_50_ value of 650 nM in the 48H direct virus inhibition assay and 1.51 M in the 72H CPE assay (Figure 2G and H). Krähling et al.^7^ and others^8^, ^9^ demonstrated that in the infected cells, SARS‐CoV activates PKR, leading to sustained phosphorylation of eIF2α and suppression of host translation. Thus, our data present EIF2AK2 kinase as a viable drug target for therapies to target viral translation and also to modulate the host interferon response.

**TABLE 1:** Antiviral activities of PKR inhibitor C16 in 72H cytopathic and 48H virus inhibition assays

	**Cell line**					
	**VeroE6**		**VeroE6+TMPRSS2**		**A549+ACE2**	
	**EC_50_ (μM)**		**EC_50_ (μM)**		**EC_50_ (μM)**	
**Compound**	**48H virus inhibition**	**72H CPE**	**48H virus inhibition**	**72H CPE**	**48H virus inhibition**	**72H CPE**
**C16**	**0.568**	**1.06**	**1.65**	**2.06**	**1.25**	**2.96**
**Remdesivir**	**1.39**	**1.86**	**1.45**	**1.86**	**0.153**	**0.171**
**C16+remdesivir**	**1.06**	**1.51**	**N/D**	**N/D**	**N/D**	**N/D**

*Note*: For each tested compound, the antiviral activity in VeroE6, VeroE6+TMPRS22 and A549+ACE2 cells is indicated by 50% effective concentration (EC_50_) values (in micromolar). The values were gained from the 10‐point concentration‐response assays. N/D, not done

**FIGURE 2:**
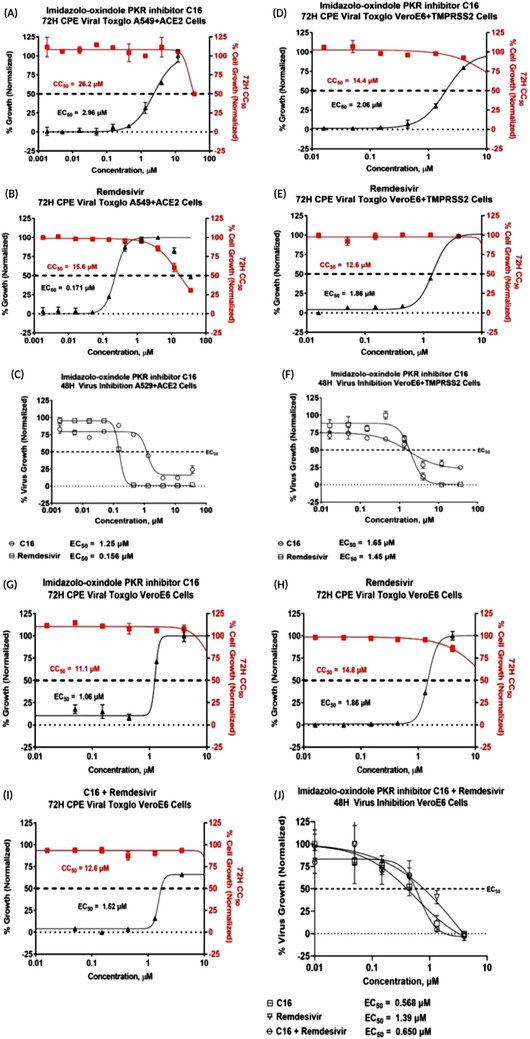
The in vitro antiviral dose‐response screening results of the EIF2AK2 inhibitor compound C16. Inhibition of SARS‐CoV‐2 replication was tested in various cells by developing CPE via ATP luminescence at 72H (solid shapes) or direct virus inhibition at 48 h (empty shapes). For the 48 h direct virus inhibition, %virus growth was normalised to the infected + DMSO only wells (max) and uninfected + DMSO only wells (min). For the 72 h CPE/ATP Luminescence assay, cell growth was normalised to the cells+DMSO control wells (Max) and Cells+DMSO+virus only (Min) wells for EC_50_ calculations (black triangles) and Cells+DMSO control wells (Max) and Media+DMSO control wells (Min) for the CC_50_ calculations (red squares, cytotoxicity). The 48H and 72H EC_50_ assays were performed at the CDI biosafety level 3 laboratory. (**A–C**) A549 human lung cells overexpressing ACE2 were treated with either C16 or remdesivir and infected with SARS‐CoV‐2 infectious clone reporter strain. C16 by itself was found to have relatively high potency in the 48H direct virus inhibition assay (EC_50_ = 1.25 μM) and the 72H ATP luminescence assay measuring cytopathic effect (EC_50_ = 2.96 μM) with low cytoxocity (CC_50_ = 26.2 μM). (**D–F**) VeroE6 African Green monkey kidney cells overexpressing TMPRSS2 were treated with either C16 or remdesivir and infected with SARS‐CoV‐2 infectious clone reporter strain. The 48H direct virus inhibition EC_50_ was 1.65 μM and the 72H CPE EC_50_ was 2.06 μM in C16 treated VeroE6+TMPRSS2 cells with low cytoxocity (CC_50_ = 14.4 μM). Similar results were observed in remdesivir treated cells. (**G–J**) Treatment of C16 on VeroE6 cells infected with SARS‐CoV‐2 shows C16 had improved potencies relative to remdesivir. VeroE6 cells treated with C16 had EC_50_ values of 0.568 and 1.06 μM in the 48H direct virus inhibition and 72H CPE assays, respectively. The combination treatment with C16 and remdesivir was also evaluated in VeroE6 cells. The EC_50_ was 650 nM in the 48H direct virus inhibition assay and 1.52 μM in the 72H CPE assay. In the remdesivir treated VeroE6 cells, the EC_50_ was 1.39 and 1.86 μM in the same assays.

To understand the association of the host EIF2AK2 hub interactions with the SARS‐CoV‐2 proteins, we mapped the interaction of EIF2AK2 with the viral SARS‐CoV‐2 Nucleocapsid protein (N) interactions based on physical evidence (BioGrid database) correlating to our identified significantly up‐ and downregulated differentially expressed genes. Notably, we found that the N protein interacts with EIF2AK2 (Figure 3A), in addition to a group of interferon‐induced proteins (IFIT2, IFIT3, IFIT5) (Figure 3B), which contribute to the dysregulation of innate immunity of the infected COVID‐19 patients. Our structural modelling analysis found that the C16 compound binds at the SARS‐CoV‐2 N protein nucleotide‐binding pocket NTD domain (Figure 3C). Notably, Lin et al.^10^ reported a cocrystal structure of HCoV‐OC43 coronavirus N protein inhibitor PJ34 targeting the same high sequence similarity nucleotide‐binding pocket (PDB:4KXJ). Thus, we speculate that C16 could act as a dual inhibitor of viral N protein and host EIF2AK2. Blocking the formation of the N protein interactome can potentially inhibit viral protein replication and protein synthesis and restore the IFN pathway.

**FIGURE 3:**
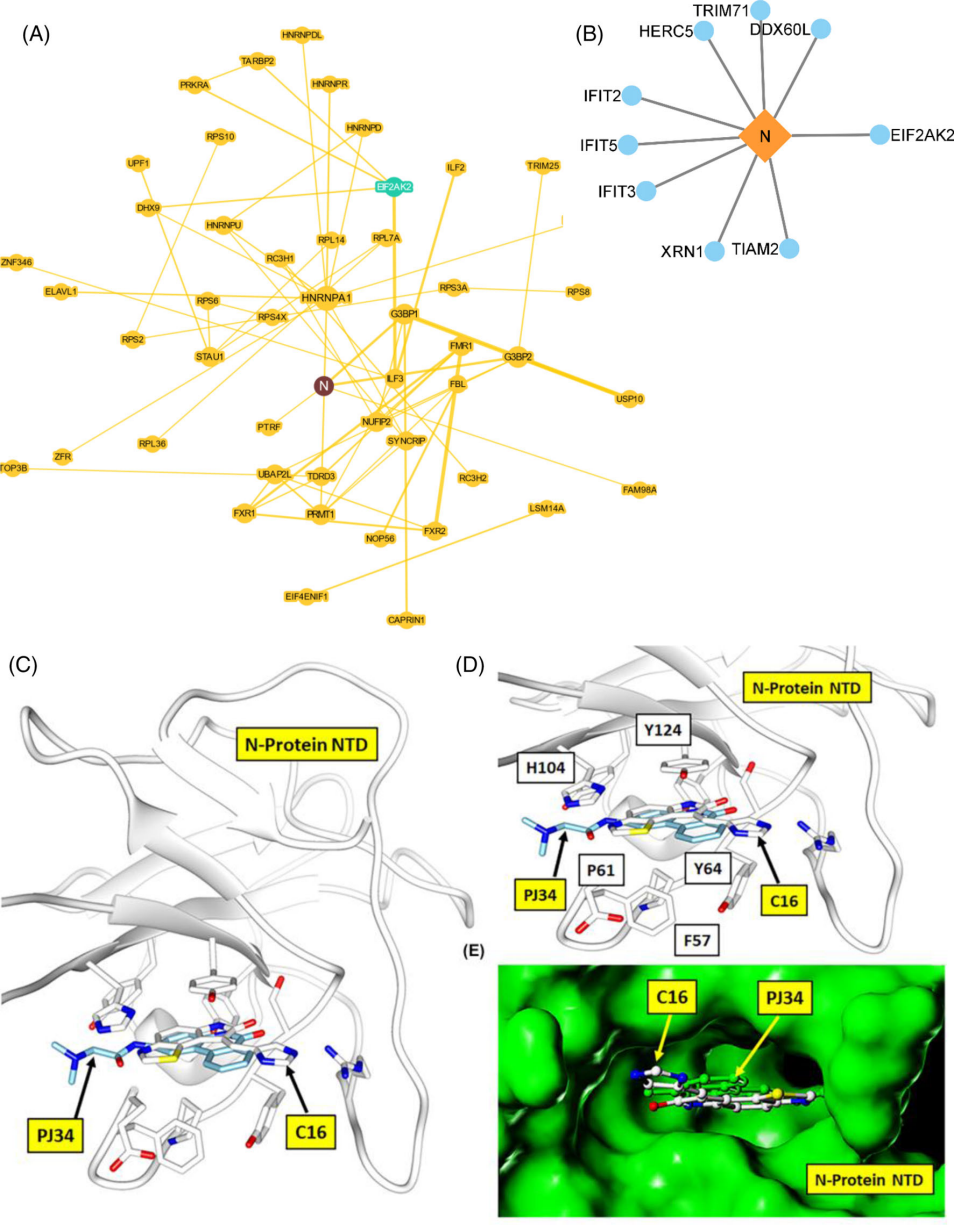
*SARS‐CoV‐2‐host protein interaction map and s*tructural modelling of the N‐Terminal Domain (NTD) of N protein in complex with C16. (**A**) SARS‐CoV‐2‐host interaction BioGRID map with mapping is centred on N protein interaction. The interaction map was obtained from the COVID‐19 patient samples RNASeq profiling. **(B)** SARS‐CoV‐2 N protein interaction with upregulated host proteins. (**C**) The structural model of N protein, NTD (the nucleotide‐binding pocket) in complex with C16 compound (carbon atom coloured white) with binding site residues interacting with the C16 is shown as a stick model. The HCoV‐OC43 coronavirus N protein inhibitor PJ34 (indicated with carbon atom‐coloured cyan) is structurally similar to C16 and binds at the same NTD nucleotide‐binding pocket, justifying the hypothetical structural model of the N protein complex with C16. (**D**) The close‐up view of the N protein and C16 structural complex model. The C16 is locked tightly into the nucleotide pocket by hydrogen bond interaction of the dihydropyrrole ring and multiple stacking interactions of the N protein residues F57, P61, Y64, H104 and Y124, typical of any flat compound interaction. (**E**) The molecular surface view (coloured green) of N protein in complex with C16 and PJ34 compound, buried inside the N protein nucleotide‐binding pocket.

This study has limitations. The availability of data at the time of analysis limited the scope of our study – namely, the lack of comprehensive clinical annotations regarding treatment or disease severity and the presence of low‐quality sequencing data. Information about drug toxicity, efficacy and clinical applicability is needed to further validate the drugs, compounds, and other therapeutics identified in our analysis.

## CONCLUSION

In this study, we identified EIF2AK2 as a potential antiviral target. In multiple in vitro assays, we demonstrated that EIF2AK2 inhibitor compound C16 is a potent antiviral that could combat SARS‐CoV‐2 infection. We also found that C16 binds to an EIF2AK2 hub interacting protein, the SARS‐CoV‐2 N protein, thus could act as a dual inhibitor, conferring strong SARS‐CoV‐2 antiviral activity. Our results suggest new potential targets that have not been well‐characterised that warrant further investigation. These host therapeutic targets can help guide drug discovery efforts towards those most indicated by COVID‐19 disease signatures. While many studies have focused on potential therapeutics to target viral proteins, our analysis of important host factors contributing to disease pathogenesis provides necessary insight for more patient‐focused therapeutic strategies based on host factors and disease severity.

## AUTHOR CONTRIBUTIONS

Sivanesan Dakshanamurthy: conceptualisation, methodology, supervision, investigation, writing – reviewing and editing. Sidharth Jain: methodology, investigation, data curation, writing – reviewing and editing. Samantha Rego: methodology, investigation, data curation, writing – reviewing and editing. Steven Park: methodology, investigation, writing‐ reviewing and editing. Yiran liu: visualisation, investigation, writing – reviewing and editing. Simone Parn: investigation, visualisation, writing‐ reviewing and editing. Kush Savsani: investigation, visualisation, writing – reviewing and editing. David Perlin: investigation, writing – reviewing and editing.

## CONFLICT OF INTEREST

The authors have no conflict of interest to declare.

## Supporting information

Supplementary Material

Supplementary Table 1.xlsx

Supplementary Table 2.xlsx

Supplementary Table 3A.xlsx

Supplementary Table 3B.xlsx

Supplementary Table 4A.xlsx

Supplementary Table 4B.xlsx
