# Supplementary material for: RNASeq profiling of COVID19‐infected patients identified an EIF2AK2 inhibitor as a potent SARS‐CoV‐2 antiviral
Source: Clin Transl Med. 2022 Nov 2;12(11):e1098. doi: 10.1002/ctm2.1098 (PMC9627224; doi:10.1002/ctm2.1098)
Supplement: Supplementary file 1 — Supplementary Material [file CTM2-12-0-s002.docx]

Supplementary Material

**DETAILED MATERIALS AND METHODS**

**Acquisition and Processing of GEO Data**

To understand the host transcriptomic changes that occur upon SARS-CoV-2 infection, we first gathered relevant publicly available gene expression datasets. We queried the NCBI Gene Expression Omnibus (GEO) database^1^for RNAseq datasets, focusing on patient samples obtained from the pulmonary tract (**Supplementary Figure S1**). We found four datasets (**Supplementary Table S1**) that satisfied the query criteria and obtained FASTQ-level data for each sample. Using the standard RNAseq pipeline in *Partek Flow* software, v10.0.^2^, samples were processed, and gene-level counts were quantified. For GSE152075, FASTQ data could not be collected due to patient privacy concerns, but gene-level counts were provided, which were used for further analysis.

**Differential Expression Analysis**

Data collected from GEO were processed using the Gene Set Analysis algorithm in *Partek Flow* software, v10.0., to identify differentially expressed genes in each experiment. Samples were labeled based on the presence or absence of detected SARS-CoV2, and only respiratory samples were compared. Differentially expressed genes from each dataset were identified at a threshold of FDR < 0.05, and up-and down-regulated genes were defined using a fold-change cutoff of ∓2 (**Supplementary Tables 1 and 2**). Genes found as upregulated in two or more experiments were selected for further analysis.

**Pathway Analysis and Interaction Mapping**

Differentially expressed genes identified by Gene Set Analysis in *Partek Flow* software, v10.0., for each dataset were separated into upregulated and downregulated based on fold change and uploaded into Ingenuity Pathway Analysis (IPA)^3^. To identify the significant pathways implicated by the patterns of differential gene expression in each dataset, we ran the analysis with thresholds at FDR < 0.05 and fold change ∓2 in IPA separately for upregulated and downregulated genes for each dataset (**Supplementary Tables 1, 2, 3, 4**).

The comparison analysis in IPA was used to compare the upregulated pathways to the downregulated pathways across datasets. Pathway diagrams for pathways identified as most significantly up-or down-regulated in both datasets were analyzed for drug and gene interactions and downstream effects of the observed differential gene expression.

**Identifying Drug-Gene Interactions**

Differentially expressed genes were queried in Drug Gene Interaction Database (DGIdb) to identify existing drugs with experimentally determined associations with our DEGs^4^. Any genes with known drug interactions were annotated using DGIdb. Drugs were also annotated based on known action (e.g. inhibitor, antagonist), and interaction with SARS-CoV-2^5^ (**Supplementary Table 4**).

**Molecular Modeling of C16-SARS-CoV-2 Structural Complex**

The X-ray cocrystal structure of the HCoV-OC43 coronavirus N protein inhibitor PJ34 targeting the same high sequence similarity nucleotide-binding pocket (PDB:4KXJ, NTD domain) was used for the molecular docking simulations of C16 against SARS-CoV-2 N protein. The SARS-CoV-2 N protein structure was energy minimized with AMBER with SANDER default parameters.^6^ Docking experiments were performed using the Glide module of the Schrodinger molecular modeling software package (Schrodinger, Inc., USA) with the number of solution conformations set to 60. The best-docked geometry of C16 was visually analyzed, and energy-minimized with the SANDER module of AMBER.^6^

**Molecular Dynamics Simulations**

The final structural model of the C16 – SARS-CoV-2 N protein complex by relaxing the structure complex using the flexible molecular dynamics simulations. The molecular dynamics simulations were performed using the AMBER 20 simulation package.^6^ The general amber force field^7^and root-mean-square deviation charge models^8^ were utilized and other parameters were set to default. Molecular dynamics simulations performed in the microcanonical ensemble at constant-energy, constant-volume ensemble (NVE) consisted of an initial equilibration of 200 ps followed by a production run of one nanosecond simulation at temperature of 300 K. The final structural complex obtained at the end of the production run simulations was further subjected to 10000 steps of steepest descent energy minimization followed by conjugate gradient energy minimization.

**Cells and Cell Cultures**

VeroE6 cells were purchased from ATCC (Manassas, VA). VeroE6+TMPRS22 was purchased from Sekisui Xenotech (Kansas City, KS), and A549+ACE2 was purchased from Genscript Biotech (Piscataway, NJ). All cells were kept in Dulbecco's Modified Eagle's Medium (DMEM) (ATCC), up to 20% USDA sourced Fetal Bovine Serum (Thomas Scientific, Swedesboro, NJ), 1x Antibiotic/Antimycotic solution (ThermoFisher, Waltham, MA). The cell was passaged by gentle disassociation with TrypLE Express reagent (Thermo Fisher) and washed with Cell Culture Phosphate Buffered Saline (1X) (Corning Life Sciences, Tewksbury, MA).

**72H Cytopathic Assay for EC_50_and CC_50_determination**

A well-established ATP luminescence assay that is highly correlative with SARS-CoV-2 cytopathic effect was used to assess the antiviral potency and toxicity of the leads^9,10^. A 10-point concentration-response assay was performed to determine EC_50_ (effective concentration) and CC_50_ (Cytotoxic concentration) values for each compound. Final screening concentrations range from 36 to 0.018 μM (three-fold serial dilution) with a final DMSO (Millipore Sigma Burlington, MA) concentration of 0.5%. VeroE6, VeroE6+TMPRSS2 and A549+ACE2 cells were seeded in Corning 3610 opaque 96-well clear bottom plate (1×10^4^cells per well) in DMEM containing up to 20% FBS + 1x antimycotic/antibiotic solution using a Biotek EL-406 washer/dispenser (Agilent/Biotek Winooski. VT). Test compounds were added to each well using a Tecan D300e dispenser (Tecan US, Morrisville, NC), and cells were pre-treated for two hrs then mixed with an infectious SARS CoV-2 virus reporter construct icSARS-CoV-2-mNG expressing green fluorescence (courtesy K. Plante, UTMB) at a MOI of up to 0.1 using the Integra Viaflow 96 channel pipette (Integra Biosciences Hudson, NH). The final volumes of the assays were 0.1 ml (96 wells). Cells were monitored visually at 48- and 72- hours post-infection by phase-contrast microscopy for changes in adherence and morphology, and a final quantitative read-out was established from luminescent units produced by the addition of Viral ToxGlo™ (Promega Madison, WI) measuring ATP. Luminescence was detected using a Tecan Mplex D200 multimode plate reader. Cell growth was normalized to the cells+DMSO control wells (Max) and Cells+DMSO+virus only (Min) wells for EC_50_ calculations and Cells+DMSO control wells (Max) and Media+DMSO control wells (Min) for the CC_50_ calculations. EC_50_ assays were performed at the CDI biosafety level 3 laboratory.

**48H Virus Inhibition Assay for IC_50_determination**

Direct virus inhibition of the infectious icSARS-CoV-2-mNG reporter virus was performed using an integrated fluorescent detection and high content imaging cytometer. Direct virus inhibition with this reporter virus for assessing antiviral activity has been previously published and used in multiple screening applications^11^. The same 10-point dose response with final screening concentrations of as described in the 72H cytopathic assay was used. Cells were seeded in a corning 3603 back 96 well plate, treated then infected as described above.  After 48h, cells were stained with NucRed™ Live 647 ReadyProbes™ Reagent (Thermo Fisher), fixed in 10% formalin (Thermo Fisher), washed twice in sterile PBS, and counted by automated imaging using a Nexcelom Celigo imaging cytometer (Nexcelom Bioscience Lawrence, MA).  Virus fluorescence signal was normalized to the NucRed stained live cells in each well. Virus expression was normalized to the cells+DMSO control wells (Min) and Cells+DMSO+virus only (Max) wells. Assays were performed at the CDI biosafety level 3 laboratory.

**Statistical Analysis**

Statistical analysis of gene and pathway analysis was performed by IPA which determines the significance of the association between the data set and the canonical pathway based on the ratio of the number of proteins from the data set that map to the pathway divided by the total number of proteins that map to the canonical pathway, as well as by the p-value of the association, calculated by a right-tailed Fisher’s Exact Test. A threshold of p < .05 and FDR <.05 were retained for significance.

**DETAILED RESULTS**

**RNASeq differential gene expression analysis**

We queried the GEO for bulk RNA-seq expression data from human patients infected with COVID-19. Only studies with human lung or respiratory tract samples obtained from patients infected by COVID-19 were utilized. Our search of the GEO database revealed four RNAseq datasets that met the inclusion criteria for the study (**Supplementary Figure S2**). The largest set of samples was selected from GSE152075, which provided expression data from 484 patients. Autopsy samples were also included from recently deceased COVID-19 patients, as seen in GSE150316. Lung biopsy samples from GSE147507 were also used, although only two human patient samples were included in this dataset.  One dataset (GSE151803) was excluded from analysis due to poor read quality and lack of patient negative controls. We identified 509 eligible patient RNA-seq samples from GEO (**Supplementary Table S1**).

Each dataset was individually analyzed for differentially expressed genes. RNAseq analysis was performed on the three datasets using the *Partek Flow* genomics suite with a standard RNAseq pipeline. Differential gene expression analysis was performed using the Gene Set Analysis tool. Differentially expressed genes (DEGs) were identified by comparing samples from SARS-CoV-2 infected patients and uninfected controls in each of these datasets. Differentially expressed genes in COVID-19 patients were identified with a significance threshold of FDR < 0.05, and significantly up-or down-regulated genes were defined as having an absolute log fold change > 2. This analysis identified 457 significantly upregulated genes and 2380 significantly downregulated genes in GSE152075 (**Supplementary Figure S1a, Supplementary Table 1**). Two hundred three significantly upregulated genes and 55 significantly downregulated genes were identified from GSE147507 (**Supplementary Figure S1b, Supplementary Table 2**). Using our parameters, only one significant DEG was identified from GSE150316, so this dataset was excluded from further analysis (**Supplementary Figure S1c**).

Next, two datasets, GSE152075 and GSE147057, were further analyzed for commonly up-and down-regulated genes through Comparison Analysis in IPA, which identified 67 commonly upregulated and 12 commonly downregulated genes (**Supplementary Table 3**). Several ribosomal proteins were found to be downregulated in both datasets. In both datasets, many interferons, cytokines, and chemokines were upregulated, reflecting an activated inflammatory immune response in COVID-19 patients.

**RNASeq differential pathway expression analysis**

Core analysis of the differentially expressed genes in IPA identified the most significant genes and pathways involved in differential expression patterns in each dataset. The protein products of these genes and respective pathways represent potential therapeutic targets for COVID-19 treatment, as they are likely most functionally involved in COVID-19 pathogenesis. Upregulated genes may be targeted by inhibition, while downregulated genes may be targets of agonism. IPA analysis highlighted several key pathways and proteins that have already been well-described in the context of SARS-CoV-2 pathogenesis, including the ACE2/TMPRSS2 surface receptors used for viral entry and interferon response, and host translation machinery. Using the Drug-Gene Interaction Database (DGIdb) and IPA pathway annotations, the most significant differentially expressed genes were also mapped to existing pharmaceutical agents **(Supplementary Table 4, Supplementary Figure S3)**. Many of these pathways are currently being targeted in ongoing clinical trials by drugs identified in our analysis, with some showing promising preliminary results, such as barcitinib^12^.

Analysis of the SARS-CoV-2 replication cycle identified key host genes involved in viral replication (**Supplementary Figure S3**). The ACE2 receptor used by SARS-CoV-2 for entry into host cells and the replication complex were downregulated in one dataset. Downregulation of ACE2 is unique in this dataset, as other studies have found ACE2 upregulation post coronavirus infection (Zhuang). Confirmation of the validity of these findings can be conducted by performing similar analysis on other patient datasets. TMPRRS2, a serine protease also used by SARS-CoV-2 for entry, was upregulated in one dataset, and tubulins were upregulated in both datasets. The drug-gene interaction analysis through DGIdb revealed multiple existing compounds with the potential to target host mechanisms of viral proliferation, specifically viral entry, replication, and assembly within host cells (**Supplementary Table 4**). Drugs, such as ribavirin and colchicine, have been reported by previous studies and are currently being tested or are in use to treat COVID-19 patients^13,14^, verifying the importance of our results. Other compounds we identified with relevant targets have not been extensively studied in COVID-19 but warrant further investigation (**Supplementary Table 4**). Other identified compounds have been shown to be ineffective in clinical trials, such as hydroxychloroquine. Still, they point to potential therapeutic opportunities and underlying biological mechanisms that may drive further drug development efforts.

**EIF2AK2 is identified as a potential therapeutic target of SARS-CoV-2**

Our analysis shows that the interferon signaling pathway was entirely upregulated, revealing many key genes responsible for activating the interferon response in SARS-CoV-2 infection (**Supplementary Figure S4**). Janus kinase/signal transducers and activators of transcription (JAK/STAT) are also significantly upregulated, as is an expression of the JAK/STAT signaling pathway. The DGIdb interaction analysis identified many compounds with the potential to modulate these upregulated key activators of the host interferon response. Many of these drugs and compounds interacting with the interferon signaling pathway have been studied in COVID-19 clinical trials. Inhibition of the JAK/STAT pathway can potentially combat the hyper inflammation seen in COVID-19^15^, and the effect of JAK inhibitors on COVID-19, including multiple identified in our analysis, are being studied in clinical trials (RUXCOVID; TOFA-CoV-2. Baricitinib has shown particularly promising results in clinical trials^16^. Other studies have also suggested the potential role of anti-TNF therapy in treating COVID-19, and some tumor necrosis factor (TNF) inhibitors such as infliximab and adalimumab are being studied in clinical trials^17^.

Of particular interest from the comparison analysis, the eukaryotic initiation factor 2 (eIF2) signaling pathway was among the most downregulated pathways (**Supplementary Figure S4**, **Supplementary Tables 1c and 2c**), which has been shown to be involved in viral replication and transcription of viral genomic material. At an individual gene-level analysis, multiple ribosomal proteins were significantly downregulated in each dataset (**Supplementary Tables 1 and 2**). In addition to changes in expression, as seen in our study and others^18^, there are also notable changes in the phosphoproteomic landscape of hosts^19^. This is also consistent with our independent large-scale patient samples of single- cell and bulk RNASeq data analysis followed by mapping downregulated host genes with the SARS- CoV-2 nucleocapsid protein using the BioGRID COVID 19 viral-host interactions (**Supplementary Figure S5**). We show that the activation of eIF2α leads to translational shut-down.

**DISCUSSION**

Our COVID19 patient RNASeq analyses identified known pathways and proteins involved in SARS-CoV-2 pathogenesis. For example, ACE2 and TMPRSS2, receptors known to be used by SARS-CoV-2 for cell entry, were significantly differentially expressed in our analysis of COVID-19 patients^20^.Many of the upregulated genes identified in our study and others are implicated in interferon response and chemokine/cytokine signaling, suggesting their importance as potential therapeutic targets for COVID-19 treatment. Viral-host interactions may also occur between viral proteins and the interferon-response signaling pathway, especially as SARS-CoV-2 has been shown to modulate interferon signaling using ORF1ab^21^. Further studies of the virus-human interactome may help elucidate the mechanism of this modulation and may reveal new therapeutic strategies for COVID-19 and other viral-borne illnesses. Of particular interest, JAK/STAT signaling is known to occur downstream of the interferon-response signaling pathway, which is significantly differentially expressed in our analysis. Concurrent to these findings, recently, the JAK inhibitor Barcitinib is currently issued an Emergency Use Authorization (EUA) to permit the emergency use for the treatment of coronavirus disease 2019 (COVID-19).

Our results are also concordant with the clinical presentation of COVID-19. Both datasets also had significant upregulation of many cytokines and the chemokine signaling pathway, suggesting an activation of the inflammatory response in COVID-19 patients. Excessive release of cytokines by the dysregulated immune system is characteristic of severe COVID-19 infection, leading to a cytokine release storm^22^. This leads to acute respiratory distress syndrome (ARDS), a major cause of morbidity and mortality in COVID-19 patients, making management of excessive cytokine release a critical treatment strategy for improving patient outcomes^22^. Our results affirm the important role of cytokines in COVID-19 pathogenesis and suggest specific cytokine drug targets, such as CXCL10 and TNFSF13B, and potential therapies whose effect on COVID-19 should be studied further (**Supplementary Table 4**).

Several target genes, pathways, and potential therapies identified by our analyses have not been studied extensively in COVID-19. While other studies have characterized the importance of the interferon response in COVID-19 pathogenesis, our results suggest specific key genes in this pathway affected in COVID-19 patients and specific compounds that can target these genes that have not been well-studied. The critical role of JAK/STAT signaling in interferon response is also suggested by our results but has also not been extensively studied in the context of COVID-19. Many of the JAK and STAT inhibitors we identified as potential drug candidates are already under study in COVID-19 treatment^23,24^. Our results help illuminate the mechanisms underlying their impact on the host immune response in SARS-CoV-2 infection and further validate the importance of studies of these potential therapies. We also found TLR4 inhibitors as novel therapeutics for study in COVID-19 treatment. Although the effect of TLR4 inhibition in COVID-19 has not been studied, our results suggest the potential therapeutic efficacy of such a strategy. The significance of TLRs in COVID-19 pathogenesis displayed by our results is supported by evidence that SARS-CoV-2 spike proteins interact with extracellular domains of multiple TLRs, including TLR4, demonstrating their important role in SARS-CoV-2 infection^25^.

Analysis of significantly downregulated genes revealed suppression of many ribosomal proteins in both datasets analyzed, suggesting the importance of the interaction of viral proteins with host ribosomes in SARS-CoV-2 infection. Instead of entering the nucleus, SARS-CoV-2 accesses and takes over the ribosomes of the host cell directly. Yuan et al. reported that SARS-CoV-2 uses its nonstructural protein 1 (nsp1) to bind to ribosomes and suppress host cell protein production while promoting viral protein synthesis, enabling the virus to redirect host protein synthesis machinery towards viral proteins^26^. Specifically, nsp1 has been found to target the 40S, and Schubert et al. used cryo-EM to show how nsp1 binds to 40S, blocking the mRNA entry channel to inhibit mRNA binding and translation initiation^27^. This mechanism may explain why the 40S and other ribosomal proteins were found to be significantly downregulated in COVID-19 patients in the datasets we analyzed (**Supplementary Tables 1 and 2**), indicating translation inhibition consistent with the effects of nsp1 reported by Schubert et al^27^. The prevalence of decreased expression of ribosomal proteins observed in our analysis suggests the importance of this mechanism of viral infection and production in COVID-19. These findings indicate the relevance of EIF2AK2 as an upstream drug target of eIF2 signaling. Although our analysis did not include compounds that target viral proteins, nsp1 could also serve as an important target for antiviral therapy.

To understand the association of the host EIF2AK2 hub interactions with the SARS-CoV-2 proteins, we obtained the SARS-CoV-2 N protein interactions with physical evidence (BioGrid database) correlating to our identified significantly up-and-downregulated differentially expressed genes. Previous studies suggest that N protein is highly expressed during SARS-CoV-2 infection. Given its multiple functions in the viral life cycle and roles in modulating the host cellular machinery, SARS-CoV-2 N protein has been reported as a promising target for vaccine and antiviral drug development^28^. The EIF2AK2 interaction with N protein (**Supplementary Figure S6**) indicates that the EIF2AK2 is involved in the SARS-CoV-2 viral replication pathogenesis pathway (**Supplementary Figure S6**). Additionally, the direct interaction of N protein with EIF2AK2 (**Supplementary Figures S6**) and its interacting partners (IFIT2, IFIT3, IFIT5) may suggest that N protein inhibits the production of IFN by antagonizing these proteins.

The *in-vitro* experiment data suggests that C16 can reduce the viral proliferation of SARS-CoV-2 in the *in vitro* setting and that PKR inhibition may serve as a viable therapeutic strategy for the treatment of COVID-19. Our results show that C16 could combat SARS-CoV-2 infection and validate our identification of EIF2AK2 as a critical host factor in SARS-CoV-2 pathogenesis. Further, C16 administration in murine models has shown no serious side effects, specifically when monitoring body weight^29^.

**SUPPLEMENTARY TABLES**

| **GEO Accession** | **Platform** | **Study design** | **Tissue** | **Sample size** | **Infected** | **Controls** |
| --- | --- | --- | --- | --- | --- | --- |
| **GSE152075** | **GPL18573** | Examined host gene expression across infection status, viral load, age, and sex among RNA-sequencing profiles of nasopharyngeal swabs. | A nasopharyngeal swab from the upper respiratory tract | **484** | **430** | **54** |
| **GSE150316** | **GPL18573** | Autopsy samples from patients deceased due to SARS-Cov2 infection were collected for RNA-seq analysis to assess viral load and immune response. | Lung, heart, jejunum, liver, kidney, bowel, fat, skin, and marrow biopsies | 37  **(21 eligible lung tissue)** | 32  **(16)** | **5** |
| **GSE147507** | **GPL18573** | In humans, primary human lung epithelium (NHBE), lung alveolar cells (A549), and Calu-3 cells were mock-treated or infected with SARS-CoV-2, IAV, or RSV. Ferrets were mock-treated or infected with pH1N1 virus or SARS-CoV-2. Lung biopsies from two healthy participants and one COVID-19 patient were also analyzed. | Primary human lung epithelium (NHBE), lung alveolar cells (A549), Calu-3 cells, lung biopsies  Ferret nasal washes and trachea | 110  **(4 eligible human lung biopsies)** | 67  **(2)** | 43  **(2)** |
| **Total** |  |  |  | **509** | **448** | **61** |

**Table S1. Datasets from the NCBI Gene Expression Omnibus (GEO) database were included in the analysis.** Datasets were identified from the query in the GEO database as of July 28, 2020. The bolded numbers in the “Infected” and “Controls” columns represent samples that met the inclusion criteria of human samples obtained from the respiratory tract.

**SUPPLEMENTARY FIGURES**


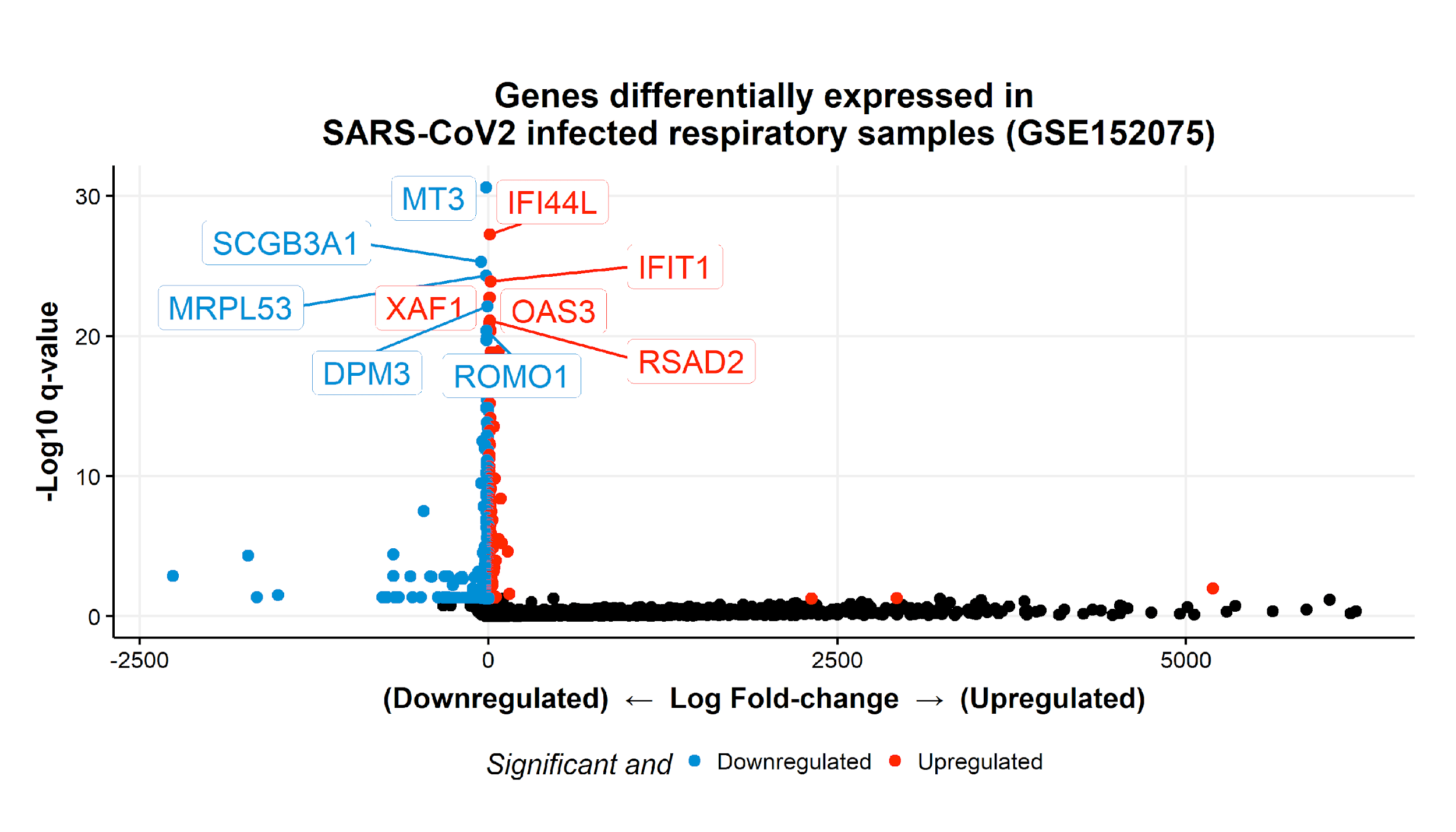

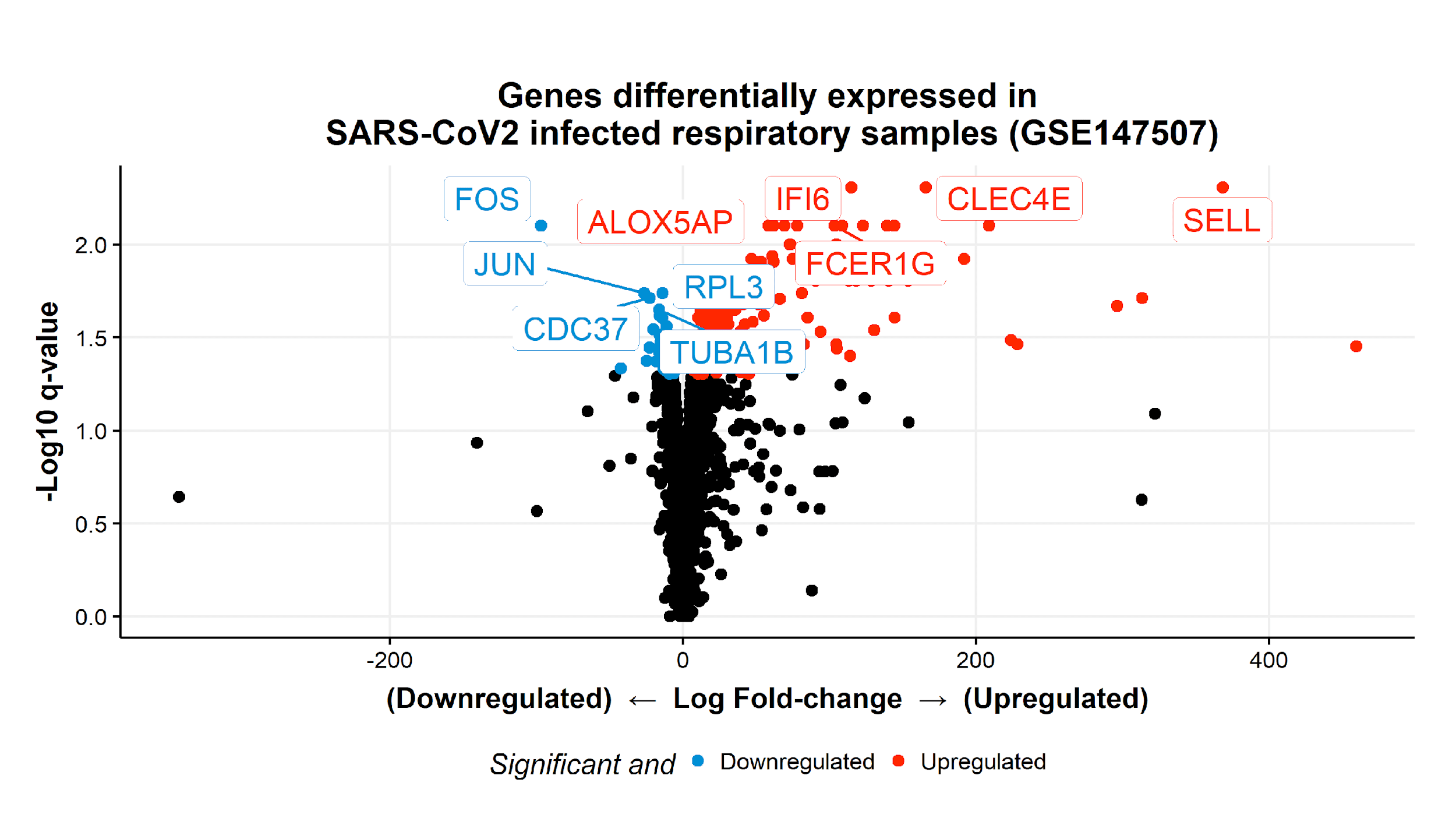


**C**

**A**

**B**

**
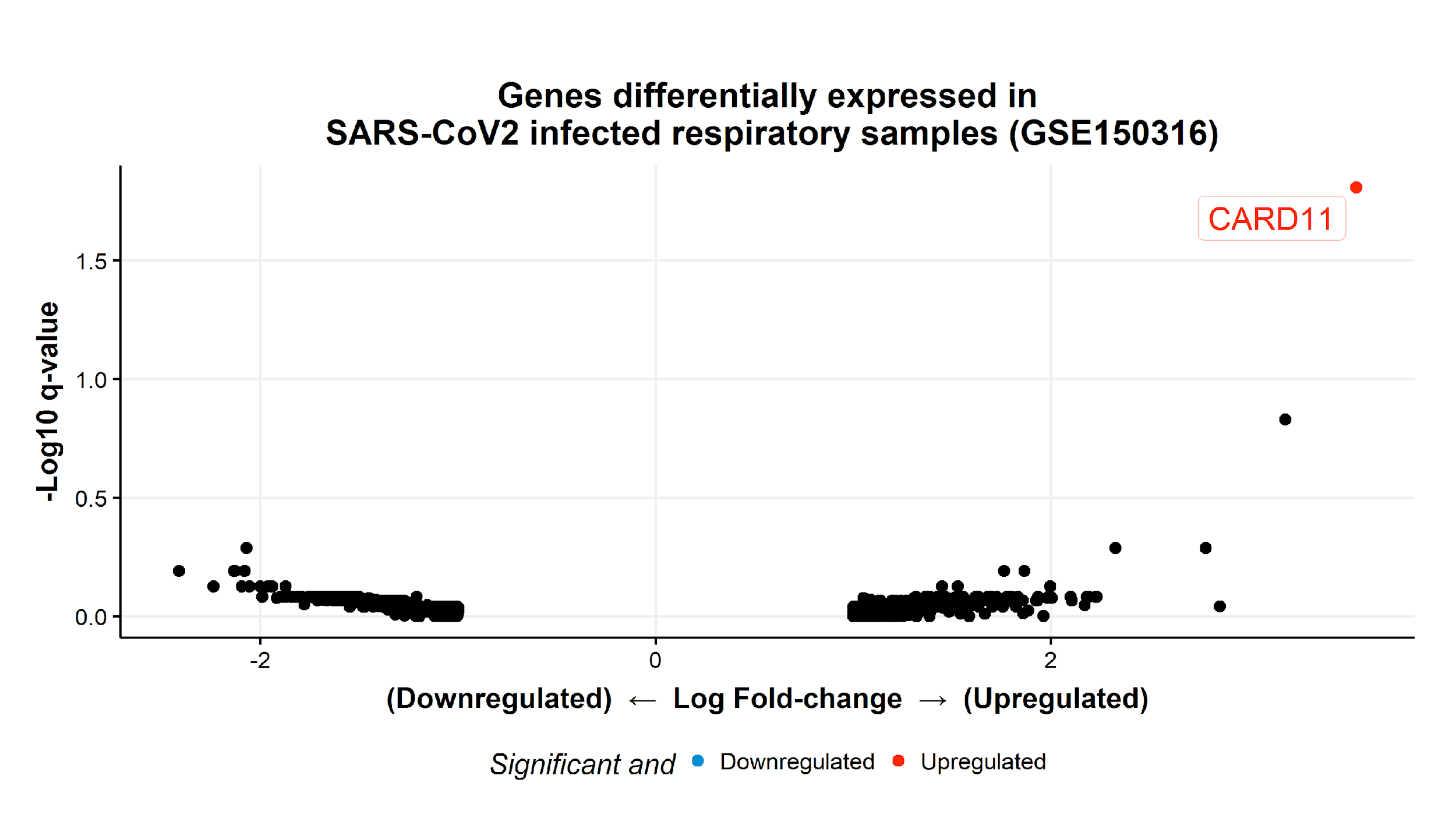
**

**Figure S1. Significantly up-and down-regulated genes in SARS-CoV-2 infected respiratory samples in each of the three analysed datasets.** Significantly upregulated genes are indicated by red, and significantly downregulated genes are shown by blue. Differentially expressed genes were identified using *Partek* *Flow* at a threshold of false discovery rate (FDR) < 0.05 and log2 fold-change >1. Volcano plots were generated using R. (**A**) Analysis of GSE152075 (n=484) found 440 significantly upregulated and 2443 significantly downregulated genes. (**B**) Analysis of GSE147507 (n=4) found three significantly upregulated and 55 significantly downregulated genes. (**C**) Analysis of GSE150316 (n=21) found one significantly upregulated gene and no significantly downregulated genes.

**Figure S2. Prisma Flow Diagram of the queried studies from the GEO database.** From Mother D, Liberati A, Tetzlaff J, Altman DG, The PRISMA Group (2009). Preferred Reporting Items for Systemic Reviews and Meta-Analyses: The PRISMA Statement. PLoS Med 6(7): e1000097. Doi:10.1371/journal.pmed1000097.


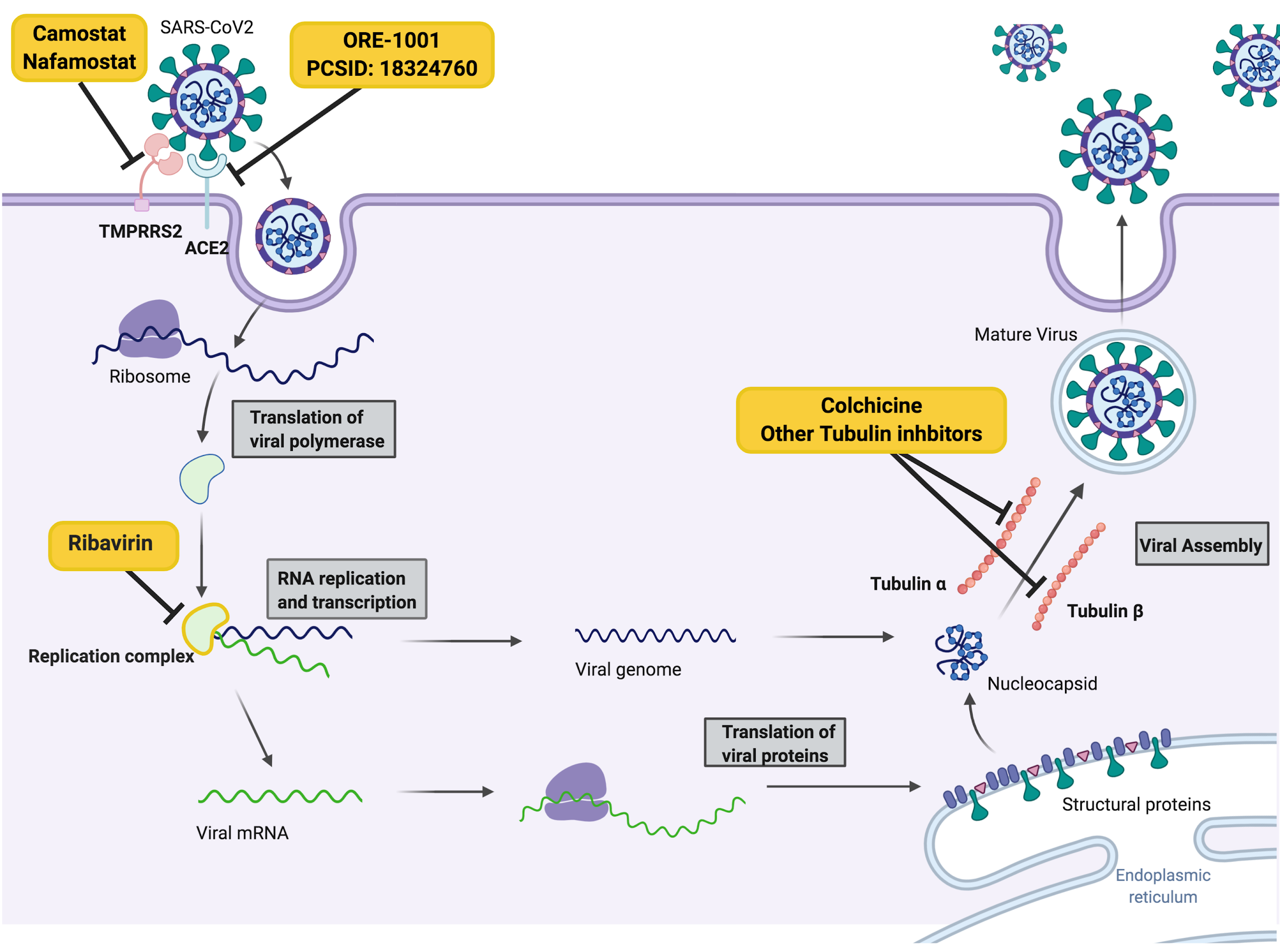

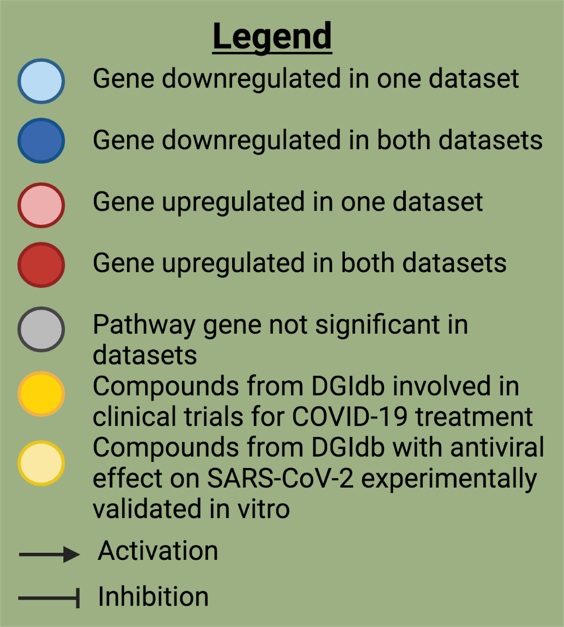


**Figure S3. Differentially expressed genes indicate potential drug targets for blocking viral entry, replication, and assembly in the SARS-CoV-2 replication cycle.** A simplified representation of the SARS-CoV-2 replication cycle was generated in [BioRender.com](https://biorender.com/) to highlight the key genes and pathways indicated as significant in our analyses, especially potential drug targets. Compounds identified from the DGIdb analysis whose effects on COVID-19 have been suggested by other studies are shown modulating their respective targets. Figure legend was also designed in [BioRender.com](https://biorender.com/)**.** Adapted from “Coronavirus Replication Cycle”, by BioRender.com (2020). Retrieved from<https://app.biorender.com/biorender-templates>. Figure legend was also designed in [BioRender.com](https://biorender.com/)**.**

A B


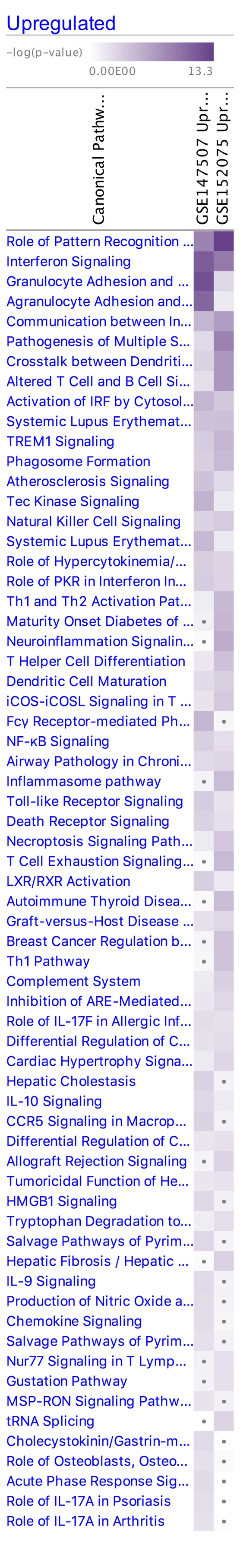

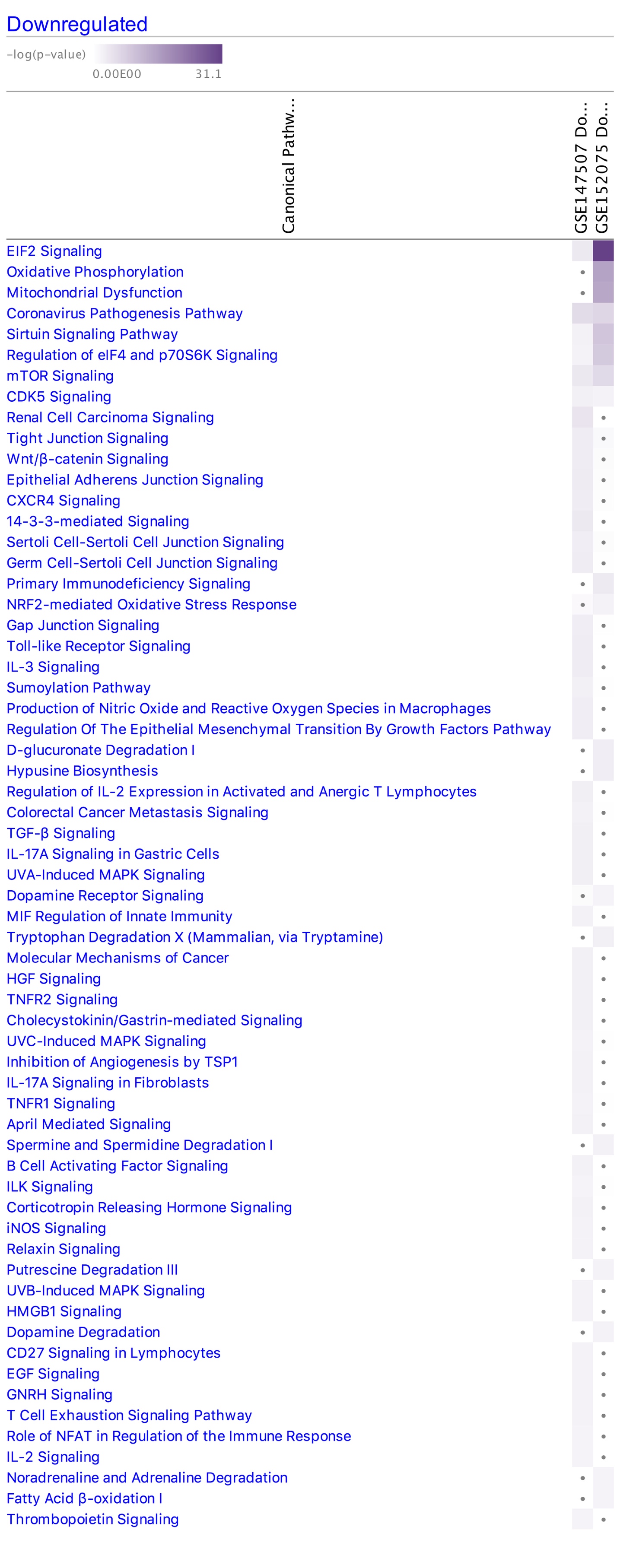


**Figure S4. The Ingenuity Pathway Analysis revealed significantly upregulated and downregulated pathways in GSE147507 and GSE152075 datasets. (A)** Upregulated pathways in the two datasets, whereas interferon signaling is among the top most upregulated signaling pathways in the COVID-19 infected samples. (**B**) Downregulated pathways in the two datasets. Our results indicate significant downregulation of subunit proteins in the EIF2 signaling pathway.


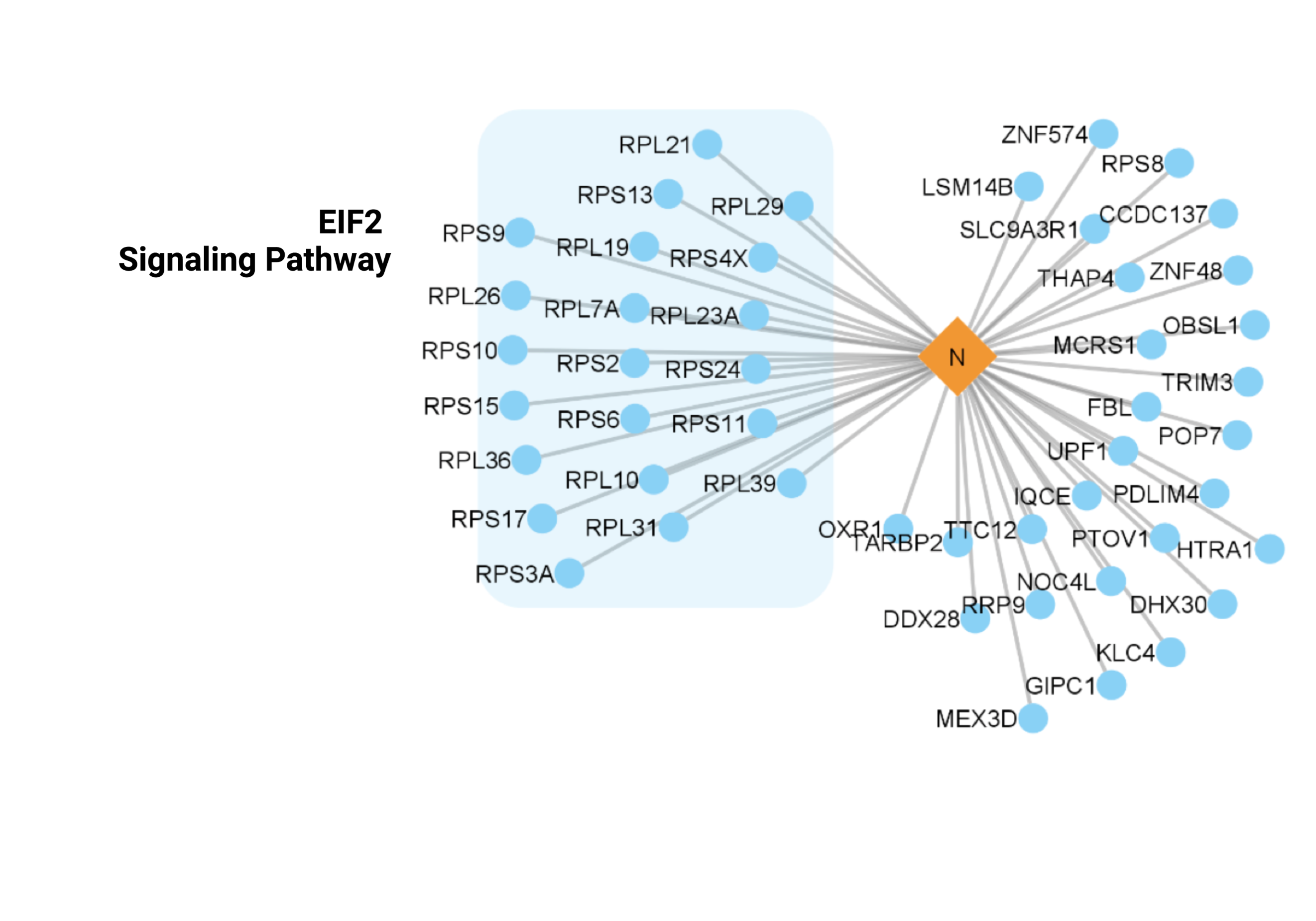


**Figure S5.** SARS-CoV-2 N protein interactions with downregulated host proteins obtained from the COVID-19 patient samples. The map was created using the BioGRID COVID 19 viral-host interactions.


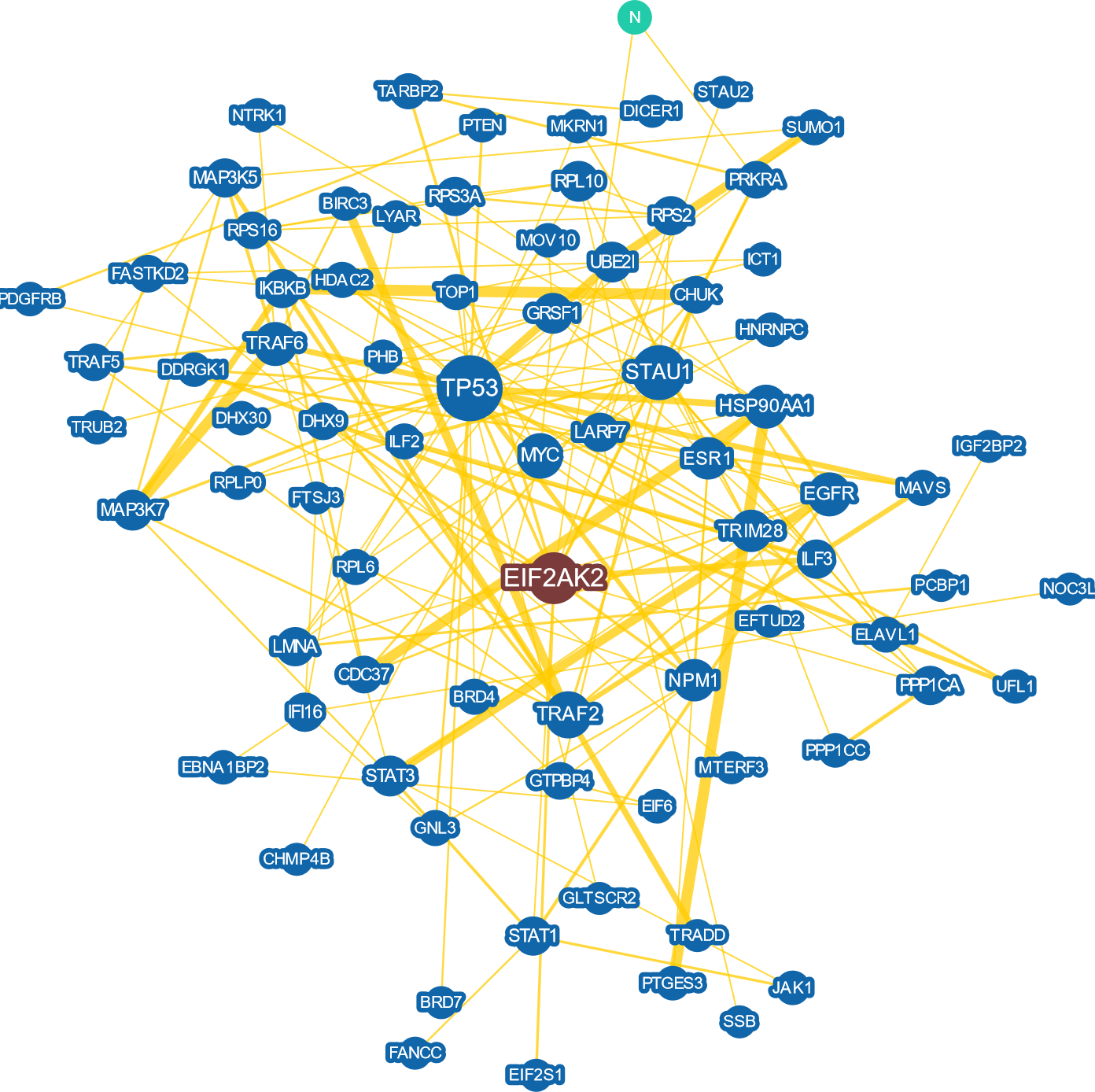


**Figure S6.** SARS-CoV-2-host protein interaction map obtained from the COVID-19 patient samples RNASeq profiling with mapping is centered on eIFaAK2 host protein.

**References**

1. Barrett, T.; Wilhite, S. E.; Ledoux, P.; Evangelista, C.; Kim, I. F.; Tomashevsky, M.; Marshall, K. A.; Phillippy, K. H.; Sherman, P. M.; Holko, M. et al. A. NCBI GEO: archive for functional genomics data sets--update. *Nucleic Acids Res*. **2013**, *41*(Database issue):D991-5.
2. Partek Inc. Partek® Flow® (Version 10.0) [Computer software]. **2020**. <https://www.partek.com/partek-flow/>
3. Causal analysis approaches in Ingenuity Pathway Analysis. *Bioinformatics.* **2014**, *30*(4), 523-30.
4. Freshour, S.; Kiwala, S.; Cotto, K. C.; Coffman, A. C.; McMichael, J. F.; Song, J.; Griffith, M.; Griffith, O. L.; Wagner, A. H. [Integration of the Drug–Gene Interaction Database (DGIdb 4.0) with open crowdsource efforts.](https://doi.org/10.1093/nar/gkaa1084) *Nucleic Acids Res*. **2020**, DOI: 10.1093/nar/gkaa1084. PMID: [33237278](https://pubmed.ncbi.nlm.nih.gov/33237278/)
5. Gordon, D.E.; Jang, G.M.; Bouhaddou, M.; Xu, J.; Obernier, K.; White, K. M.; O’Meara, M. J.; Rezelj, V. V.; Guo, J. Z.; Swaney, D. L. et al. A SARS-CoV-2 protein interaction map reveals targets for drug repurposing. *Nature.* **2020**, *583*, 459–468. Retrieved July 20, 2020, from DOI: [10.1038/s41586-020-2286-9](https://doi.org/10.1038/s41586-020-2286-9)
6. Case D, Belfon K, Ben-Shalom IY, Brozell SR, Cerutti DS, Cheatham ITE, et al. Amber Reference Manual. San Francisco: University of California; 2020.
7. Wang J, Wolf RM, Caldwell JW, Kollman PA, Case DA. Development and testing of a general amber force field. J Comput Chem 2004;25:1157-74.
8. Bayly CI, Cieplak P, Kollman PA. A well-behaved electrostatic potential based method using charge restraints for deriving atomic charges—the RESP model. J Phys Chem 1993;97:10269-80
9. Poh CM, Carissimo G, Wang B, Amrun SN, Lee CY, Chee RS, Fong SW, Yeo NK, Lee WH, Torres-Ruesta A, Leo YS, Chen MI, Tan SY, Chai LYA, Kalimuddin S, Kheng SSG, Thien SY, Young BE, Lye DC, Hanson BJ, Wang CI, Renia L, Ng LFP. Two linear epitopes on the SARS-CoV-2 spike protein that elicit neutralising antibodies in COVID-19 patients. Nat Commun. 2020 Jun 1;11(1):2806. doi: 10.1038/s41467-020-16638-2.
10. Berg MG, Zhen W, Lucic D, Degli-Angeli EJ, Anderson M, Forberg K, Olivo A, Sheikh F, Toolsie D, Greninger AL, Cloherty GA, Coombs RW, Berry GJ. Development of the RealTime SARS-CoV-2 quantitative Laboratory Developed Test and correlation with viral culture as a measure of infectivity. J Clin Virol. 2021 Oct;143:104945. doi: 10.1016/j.jcv.2021.104945.
11. Xie X, Muruato A, Lokugamage KG, Narayanan K, Zhang X, Zou J, Liu J, Schindewolf C, Bopp NE, Aguilar PV, Plante KS, Weaver SC, Makino S, LeDuc JW, Menachery VD, Shi PY. An Infectious cDNA Clone of SARS-CoV-2. Cell Host Microbe. 2020 May 13;27(5):841-848.e3. doi: 10.1016/j.chom.2020.04.004.
12. Kalil AC, Patterson TF, et al., Beigel JH; ACTT-2 Study Group Members. Baricitinib plus Remdesivir for Hospitalized Adults with Covid-19. N Engl J Med. 2021 Mar 4;384(9):795-807. doi: 10.1056/NEJMoa2031994. Epub 2020 Dec 11. PMID: 33306283; PMCID: PMC7745180.
13. Xing, J.; Shankar, R.; Drelich, A.; Paithankar, S.; Chekalin, E.; Dexheimer, T.; Chua, M.; Rajasekaran, S.; Tseng, C. K.; Chen, B. Analysis of infected host gene expression reveals repurposed drug candidates and time-dependent host response dynamics for COVID-19. *BioRxiv.* **2020**, 2020.04.07.030734, Retrieved August 10, 2020, from DOI: [10.1101/2020.04.07.030734](https://doi.org/10.1101/2020.04.07.030734).
14. Brodin, P. Immune determinants of COVID-19 disease presentation and severity. *Nat. Med.* **2021**, *27*(1), 28–33, Retrieved January 28, 2021 from DOI: [10.1038/s41591-020-01202-8](https://doi.org/10.1038/s41591-020-01202-8).
15. Satarker, S.; Tom, A.A.; Shaji R.A.; Alosious, A.; Luvis, M.; Nampoothiri, M. JAK-STAT pathway inhibition and their implications in COVID-19 therapy. *Postgrad. Med*. **2020**, *0*(0), 1–19, Retrieved January 10, 2021, from DOI: 10.1080/00325481.2020.1855921.
16. Kalil AC, Patterson TF, et al., Beigel JH; ACTT-2 Study Group Members. Baricitinib plus Remdesivir for Hospitalized Adults with Covid-19. N Engl J Med. 2021 Mar 4;384(9):795-807. doi: 10.1056/NEJMoa2031994. Epub 2020 Dec 11. PMID: 33306283; PMCID: PMC7745180.
17. Study of Adalimumab or Placebo in Patients With Mild to Moderate COVID-19 (COMBAAT) (COMBAAT). https://clinicaltrials.gov/ct2/show/NCT04705844
18. Policard M, Jain S, Rego S, Dakshanamurthy S. Immune characterization and profiles of SARS-CoV-2 infected patients reveals potential host therapeutic targets and SARS-CoV-2 oncogenesis mechanism. Virus Res. 2021 Aug;301:198464. doi: 10.1016/j.virusres.2021.198464. Epub 2021 May 29. PMID: 34058265.
19. Stukalov, A., Girault, V., Grass, V. et al. Multilevel proteomics reveals host perturbations by SARS-CoV-2 and SARS-CoV. Nature 594, 246–252 (2021). https://doi.org/10.1038/s41586- 021-03493-4
20. Hoffmann, M.; Kleine-Weber, H.; Schroeder, S.; Krüger, N.; Herrler, T.; Erichsen, S.; Schiergens, T.S.; Harrier, G.; Wu, N.; Nitsche, A. et al. SARS-CoV-2 cell entry depends on ACE2 and TMPRSS2 and is blocked by a clinically proven protease inhibitor. *Cell*. **2020**, *181*(2), 271-280.e8. Retrieved January 18, 2021, from [DOI: 10.1016/j.cell.2020.02.052](https://doi.org/10.1016/j.cell.2020.02.052).
21. Kumar, R.; Verma, H.; Singhvi, N.; Sood, U.; Gupta, V.; Singh, M.; Kumari, R.; Hira, P.; Nagar, S.; Talwar, C. et al. Comparative genomic analysis of rapidly evolving SARS-CoV-2 reveals mosaic pattern of phylogeographical distribution. Edited by Ileana M. Cristea. *MSystems*. **2020,** *5*(4), e00505-20. Retrieved January 14, 2021, from DOI: [10.1128/mSystems.00505-20](https://doi.org/10.1128/mSystems.00505-20).
22. Moore, J. B. & June, C. H. Cytokine release syndrome in severe COVID-19. *Science*. **2020**, *368*(6490), 473. Retrieved November 18, 2020, from DOI: [10.1126/science.abb8925](https://doi.org/10.1126/science.abb8925).
23. Goker Bagca, B. & Biray Avci, C. The potential of JAK/STAT pathway inhibition by ruxolitinib in the treatment of COVID-19. *Cytokine & Growth Factor Rev*. **2020**, *54*, 51–62. Retrieved January 10, 2021, from DOI: [10.1016/j.cytogfr.2020.06.013](https://doi.org/10.1016/j.cytogfr.2020.06.013)
24. Satarker, S.; Tom, A.A.; Shaji R.A.; Alosious, A.; Luvis, M.; Nampoothiri, M. JAK-STAT pathway inhibition and their implications in COVID-19 therapy. *Postgrad. Med*. **2020**, *0*(0), 1–19, [Retrieved January 10, 2021, from DOI: 10.1080/00325481.2020.1855921](https://doi.org/10.1080/00325481.2020.1855921).
25. Choudhury, A. & Mukherjee, S. In silico studies on the comparative characterization of the interactions of SARS‐CoV‐2 spike glycoprotein with ACE‐2 receptor homologs and human TLRs. *J. Med. Virol*. **2020**, 1‐9. Retrieved January 22, 2021, from DOI: [10.1002/jmv.25987](https://doi.org/10.1002/jmv.25987)
26. Yuan, S.; Peng, L.; Park, J.J.; Hu, Y.; Devarkar, S.C.; Dong, M.B.; Shen, Q.; Wu, S.; Chen, S.; Lomakin, I.B.; Xiong, Y. Nonstructural protein 1 of SARS-CoV-2 is a potent pathogenicity factor redirecting host protein synthesis machinery toward viral RNA. *Mol. Cell.* **2020**, *80*(6), 1055-1066.e6. Retrieved January 14, 2021, from DOI: [10.1016/j.molcel.2020.10.034](https://doi.org/10.1016/j.molcel.2020.10.034)
27. Schubert, K.; Karousis, E.D.; Jomaa, A.; Scaiola, A.; Echeverria, B.; Gurzeler, L.; Leibundgut, M.; Thiel, V.; Muhlemann, O.; Ban, N. SARS-CoV-2 Nsp1 binds the ribosomal mRNA channel to inhibit translation. *Nat. Struct. Mol. Biol.* **2020**, *27*, 959–966. Retrieved January 14, 2021, from DOI: [10.1038/s41594-020-0511-8](https://doi.org/10.1038/s41594-020-0511-8)
28. Liu, Wanbing et al. “Evaluation of Nucleocapsid and Spike Protein-Based Enzyme-Linked Immunosorbent Assays for Detecting Antibodies against SARS-CoV-2.” Journal of clinical microbiology vol. 58,6 e00461-20. 26 May. 2020, doi:10.1128/JCM.00461-20
29. Watanabe T, Ninomiya H, Saitou T. *et al.* Therapeutic effects of the PKR inhibitor C16 suppressing tumor proliferation and angiogenesis in hepatocellular carcinoma *in vitro* and *in vivo*. *Sci Rep* **10,**5133 (2020). https://doi.org/10.1038/s41598-020-61579-x
